# Modelling Proteasome and Proteasome Regulator Activities

**DOI:** 10.3390/biom4020585

**Published:** 2014-06-20

**Authors:** Juliane Liepe, Herman-Georg Holzhütter, Peter M. Kloetzel, Michael P. H. Stumpf, Michele Mishto

**Affiliations:** 1 Theoretical Systems Biology, Division of Molecular Biosciences, Imperial College London, London SW7 2AZ, UK; E-Mail: m.stumpf@imperial.ac.uk; 2 Institute of Biochemistry, Charité-Universitätsmedizin Berlin, 10117 Berlin, Germany; E-Mails: hergo@charite.de (H.-G.H.); p-m.kloetzel@charite.de (P.M.K.); 3 Centre for Integrative Systems Biology and Bioinformatics, Imperial College London, London SW7 2AZ, UK; E-Mail: m.stumpf@imperial.ac.uk; 4 Interdepartmental Center for Cancer Research “Giorgio Prodi”, University of Bologna, 40126 Bologna, Italy

**Keywords:** proteasome, mathematical model, 11S, 19S, non-catalytic modifier sites

## Abstract

Proteasomes are key proteases involved in a variety of processes ranging from the clearance of damaged proteins to the presentation of antigens to CD8^+^ T-lymphocytes. Which cleavage sites are used within the target proteins and how fast these proteins are degraded have a profound impact on immune system function and many cellular metabolic processes. The regulation of proteasome activity involves different mechanisms, such as the substitution of the catalytic subunits, the binding of regulatory complexes to proteasome gates and the proteasome conformational modifications triggered by the target protein itself. Mathematical models are invaluable in the analysis; and potentially allow us to predict the complex interactions of proteasome regulatory mechanisms and the final outcomes of the protein degradation rate and MHC class I epitope generation. The pioneering attempts that have been made to mathematically model proteasome activity, cleavage preference variation and their modification by one of the regulatory mechanisms are reviewed here.

## 1. Introduction

The ubiquitin-proteasome system (UPS) is a complex system responsible for the degradation of the majority of the cytoplasmic proteins. It is formed by a group of E1-E2-E3-E4 enzymes that tag target proteins with poly-ubiquitin chains, carry them into the proximity of proteasomes, where they are broken into small peptides [1,2]. Although polyubiquitination is determining the selection of the target proteins, various proteasome isoforms exist, which have degradation rates that vary from protein to protein [3,4], thus suggesting mechanisms within the proteasome *per se ipse* that regulate the protein degradation rates. The regulation of proteasome activity has a profound impact on cellular metabolic pathways (e.g., chromatin activation, transcription factor activation, RNA processing and ribosome biogenesis, aberrant polypeptide degradation, cell cycle and differentiation), on inflammation (e.g., cytokine production and signalling) and on the immune system (e.g., thymocyte selection and maturation, lymphocyte activation) [3,5].

The 20S proteasome, which is the central proteolytic machinery of the UPS, is composed of four stacked seven-membered rings (α_7_β_7_β_7_α_7_). The catalytic chamber comprises six catalytic subunits (two β1, β2 and β5 subunits) that carry out peptide-bond hydrolysis and peptide splicing [6,7,8]. Polypeptide substrates bind with the residues that are located at the N-terminal and the C-terminal sides of the cleaved residue to the non-primed and primed substrate-binding sites of the proteolytic pocket, respectively. Such binding provides the stability and the orientation of the substrate, thereby allowing the peptide-bond hydrolysis by the N-terminal Thr of the catalytic subunits [9]. In mammals, there exist different variants of the catalytic β subunits. Differential usage of these variants represents one example of proteasome activity regulation. For instance, following inflammatory stimuli, such as IFN-γ, the catalytic standard β1, β2 and β5 subunits peculiar to the standard proteasome are replaced by the immuno-subunits, β1i, β2i and β5i, in the newly synthesized immunoproteasome. The latter can carry out a variety of functions in the regulation of cellular homeostasis, cell cycle and other metabolic processes, as well as major histocompatibility complex (MHC) class I-mediated antigen presentation [3,10]. Differences in the peptide-bond cleavage preferences of standard and immune proteasomes and the implications for MHC class I epitope production have been investigated in considerable detail. Recently, we were able to demonstrate that the catalytic-subunit substitution leads to only quantitative effects and, thus, does not result in different peptide repertoires generated by standard- and immuno-proteasomes [4]. The specific activity of immunoproteasomes or its alteration has been linked to a variety of pathologies, such as neurodegenerative and autoimmune diseases [11,12,13,14].

A second way to regulate proteasome activity is given by the binding of regulatory complexes, such as 11S and 19S complexes, to the proteasome α-rings. This facilitates the peptide channelling by gate opening, thereby controlling both substrate entry and product release [15,16]. Binding of 11S and 19S complexes also induces conformational changes of the proteasome that affect the substrate binding sites and alter its cleavage preferences [17,18,19,20,21,22]. NMR spectroscopy has provided evidence that binding of the 11S complex to the α-subunits of the archaebacterium 20S proteasome triggers a wave of allosteric modifications across a network of contiguous structural regions, which reaches the β subunit (the S3 substrate-binding pocket) [21].

A third mechanism of proteasome activity regulation consists of conformational changes induced by substrate binding, creating a type of positive/negative feedback loop. It has been shown that 20S proteasomes interconvert between multiple conformations, whose relative populations are shifted upon peptide-bond hydrolysis. Indeed, the engagement of the catalytic Thr1-α amine of each active β subunit by peptide substrates is coupled to gate opening, thus resulting in a generalized positive feedback loop, leading to proteolysis [23]. In addition, peptide substrates have been shown to regulate proteasome activity by binding non-catalytic regulatory sites, although their location remains unknown [24,25,26]. Binding of fluorogenic peptides Suc-LLVY-mna and Suc-FLF-mna to non-catalytic regulatory sites leads to 20S proteasome gate opening and an increase of the cleavage rate by all three catalytic β subunits. Because the allosteric modification acts on the proteasome gate, the presence of other gate-related regulatory complexes with stronger effects (such as 11S) cover the effect of these non-catalytic modifier sites [26]. In contrast, the peptide substrate, Z-LLE-na, binds other non-catalytic regulatory sites, thereby inhibiting the activity of the β5 subunit. Such a substrate also enhances the Boc-LLR-mca degradation (mainly catalysed by β2 subunit) by binding the β1 subunit catalytic site. The latter two regulatory mechanisms are even more pronounced in the presence of 11S or 19S complexes [25]. This suggests that specific substrate peptides can regulate proteasome peptide-bond hydrolysis by trigging conformational modifications of the β rings, also independently, to an action towards gate opening. Regulatory effects have been also described by poly-ubiquitinated protein substrates on the 26S proteasome (formed by the 20S proteasome and 19S complex). Indeed, binding of the polyubiquitinated substrate, Ub_5_-MUC_4_, to the 19S complex leads to an increased degradation of short fluorogenic peptides by the 26S proteasome [27]. It has been proposed that binding of the poly-ubiquitinated substrate to the 19S regulator in conjunction with an ATP-consuming process induces a stabilization of the interaction between the 19S complex and proteasome α subunits and facilitates the access to the catalytic sites, thereby enhancing the proteolytic rate of the proteasome core [27].

## 2. The Importance of Modelling to Understand Molecular Mechanisms

**Figure 1:**
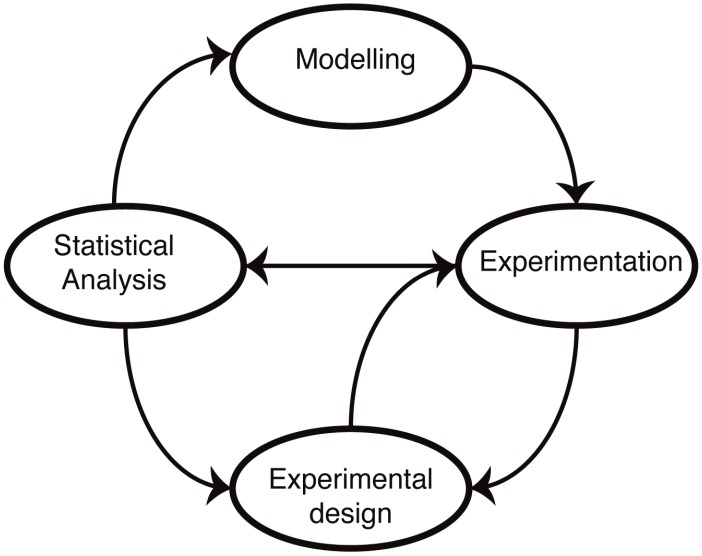
Mathematical modelling in molecular biology. Mathematical models of biological systems have to engage with experiments. Modelling prompts further experiments, and experiments allow us to parameterize and improve our models or choose from different models or mechanistic hypotheses using statistical analysis. Modelling has furthermore become an essential aspect of experimental design: not all data are informative, and investigating alternative experimental approaches *in silico* allows us to design more informative, more discriminatory experimental set-ups that result ultimately in better models.

The molecular mechanisms underlying cellular functions are typically depicted in terms of diagrams, laws, graphs, plots, relationships, chemical formulae, reaction schemes (pathways), biomolecular reactions, *etc.* Such representations form the starting point for the development of mathematical models (Figure 1). The advantage of a mathematical model is that it forces us to state our assumptions explicitly. Following this, it becomes then possible to test our understanding by either solving or simulating the resulting sets of equations. In practice, most biological systems of real-world relevance and interest are too complex to be solved exactly, and computer simulations are required to make predictions. These predictions reflect our assumptions about the mechanisms, and by comparing simulations with experimental data, we are able, at least in principle, to test our understanding. The main difference to verbal models (for example, of the type “molecule X does this when Y is present, which leads to the increase in Z”) is that the predictions are quantitative and unambiguous: there is little room for a lack of precision once a mathematical model has been invoked.

In practice, however, developing a model is only a small step. A very important, but also tedious, part of model development is the estimation of reliable values for the numerous parameters (reactions rates, binding constants, half-life of molecular complexes) entering the equations that form the model. However, before we can make predictions, we have to know these parameters. Obtaining this from of data is itself a challenge, and arguably, once these parameters are known with any degree of certainty (in the statistical sense), further analysis becomes very straightforward indeed. The increasing availability of quantitative data in cell and molecular biology has been one of the driving factors in much recent statistical research, as it has been notoriously difficult to obtain reliable parameter estimates for the mathematical models of biological systems. Recent research has shown that this need not be a problem, as long as the confidence intervals of the parameters can be evaluated [28]. In the case that a parameter cannot be inferred from experimental data, it is likely that the system is not sensitive to this parameter or that the data are not informative enough. In most cases, however, it is possible to determine parameter distributions (ranges over a parameter) that can be sufficient enough to distinguish between alternative mechanisms.

Once we have encoded our mechanistic assumptions into mathematical models, we can also: (i) compare and rank different models in light of the available data [29,30,31,32,33]; and (ii) hone the experimental setup in light of simulations of the model(s) [34,35,36]. This, in turn, enables us to improve the models and our understanding in an iterative manner.

## 3. Mathematical Models to Describe the Proteasome Hydrolysis of Short Fluorogenic Peptides

In order to understand the mechanisms of proteasome regulators, one has to understand the details of proteasome hydrolysis of peptides *per se*. One way to achieve this is by mathematically modelling the degradation rate of short fluorogenic peptides. Such peptides bind only the non-primed substrate-binding site of the proteasome. Although they are preferentially cleaved by one of the active sites, they do not mirror the cleavage preferences for peptide-bonds embedded within a polypeptide sequence [4], and thus, in the text, we avoid correlating their degradation to the so-called chymotrypsin-like, trypsin-like and caspase-like activities. Nevertheless, the usage of these substrates allowed the investigation of a simplified version of the complex proteolytic process of long polypeptides and, thus, the development of pioneering mathematical models of proteasome activity and regulation.

**Figure 2:**
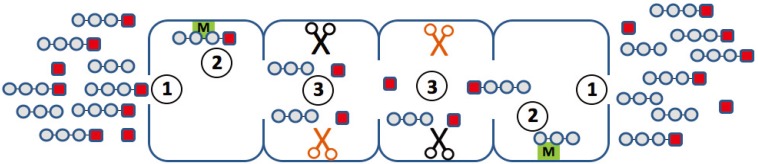
Elementary steps along the reaction path, which may determine the degradation kinetics of a small fluorogenic peptide. (**1**) Uptake and release of the peptide through the openings (gating); (**2**) binding of the peptide to non-catalytic modifier sites; (**3**) generation of the chromophore with a different efficiency at different active sites.

An ideal mathematical model of proteasomal peptide cleavage should contain a description of the separate following biophysical and biochemical steps (Figure 2):

- the substrate uptake into the proteasome chamber
- the substrate translocation inside the proteasome chamber
- binding of the substrate to the active site
- hydrolysis of the peptide bond on the active site
- release of the products out of the proteasome chamber

Furthermore, a formulation describing the influence and mechanisms of proteasome regulators should be included. However, due to the lack of suitably detailed experimental data and due to the complexity of this system, the modelling approaches so far had to focus on specific aspects of the entire hydrolysis process. In the following, we will present an overview of published models and highlight their underlying hypotheses and main findings.

**Figure 3:**
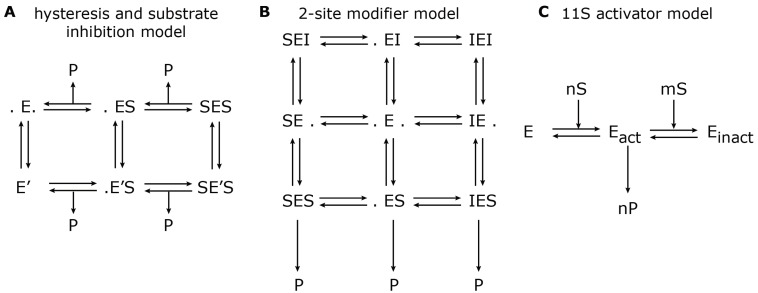
Model reaction schemes for hydrolysis of short-fluorogenic peptides. Shown are the kinetic reaction schemes to describe specific aspects of proteasome peptide hydrolysis as proposed by (**A**) Stein *et al.* [37], (**B**) Schmidtke *et al.* [24] and (**C**) Stohwasser *et al.* [15]. E denotes the proteasome (enzyme) to which a substrate, S, can bind and create a substrate-enzyme complex. A dot (.) denotes a free binding site; P denotes the resulting product. (**A**) The hysteresis model allows the transition from the standard form, E, to a modified form with a different kinetic parameter, E’. (**B**) The two-site modifier model allows for binding of a modifier molecule, I, to both binding sites, which results in changing the kinetic parameter of E. In (A) and (B), the arrows for substrate input are not shown to avoid complexity. (**C**) The 11S activator model describes the cooperative binding of n substrate molecules to the latent proteasome, E, which results in an activated proteasome, E_act_. However, further cooperative binding of m substrate molecules results in an inactive proteasome, E_inact_.

The first paper by Stein *et al.* [37] investigated the hydrolysis of the short fluorogenic peptide, Suc-LLVY-mca, over time and its dependency on the initial substrate concentration. They found that the hydrolysis of Suc-LLVY-mca by the rabbit muscle 20S proteasome exhibits two phases: an initially rapid degradation rate is followed by slower degradation setting in long before substrate depletion. The authors modelled these biphasic processes through enzyme hysteresis. They observed an inverse proportionality between the hysteresis rate and the substrate concentration. Furthermore, the authors detected a decrease of the hydrolysis rate in both initial and final phases with increasing substrate concentration, which they modelled as substrate inhibition [37]. The reaction scheme is shown in Figure 3A.

In 2000, Schmidtke *et al.* [24] used mathematical modelling in order to describe the hydrolysis rate of all three active sites under the influence of an effector (*i.e.*, Ritonavir). They found evidence for a non-catalytic modifier site, which upregulates the cleavage of the Bz-VGR-mca substrate, but downregulates the Suc-LLVY-mca degradation. The authors developed a two-site-modifier model, which they calibrated against time series data (mouse liver 20S proteasome) using a set of combinations of substrate and effector concentration. Based on the reaction scheme, the authors derived a so-called velocity equation, which describes the rate of substrate degradation at steady state. The reaction scheme and the resulting velocity equation of the two-site-modifier model is shown in Figure 3B. This kinetic model was applied to experimentally measure degradation rates of the short fluorogenic peptides, Suc-LLVY-mca, Bz-VGR-mca, Z-GGL-mca and Z-LLE-mca, under varying concentrations of the effector protein, Ritonavir. Based on the estimated model parameter, it was concluded that Ritonavir influences the hydrolysis of Suc-LLVY-mca, Bz-VGR-mca and Z-GGL-mca by binding to a non-catalytic regulator site. Ritonavir furthermore competes with Suc-LLVY-mca, Z-LLE-βNA and Z-GGL-mca, but not Bz-VGR-mca, for binding at the active site. The two effects together led to an enhanced hydrolysis of Bz-VGR-mca and an inhibited hydrolysis of Suc-LLVY-mca. Furthermore, the authors found strong self-inhibition of Suc-LLVY-mca, which could be explained by cooperative binding kinetics.

Later in the same year, Stohwasser *et al.* [15] investigated the influence of the proteasome activator, 11S, on the hydrolysis rate of the substrates, Suc-LLVY-mca, Bz-VGR-mca and Z-LLE-mca, by latent 20S proteasomes (purified from mouse liver and B8 fibroblasts) also using a kinetic model. In this model, the proteasome can exist in three states: latent proteasome, active proteasome and inhibited proteasome. Cooperative binding of substrate molecules activates the latent proteasome. However, if the substrate concentration reaches a critical threshold, the active proteasome will be inhibited. This reaction scheme (Figure 3C) was calibrated against experiments in the presence and absence of the 11S complex in order to determine the kinetic parameters that were altered. The main conclusion was that in the presence of the 11S complex, the substrate affinity to the activating site was enhanced, whereas the affinity to the inhibiting site was decreased. However, the maximum activities remained the same. These findings were consistent, independent of the substrate used. It appears that the substrate uptake and release by the proteasome are the rate limiting steps of peptide hydrolysis. The presence of the 11S complex facilitates the peptide uptake and release through the proteasome gates, which therefore results in an enhanced peptide hydrolysis.

The three different kinetic models mentioned above have in common that they treated the proteasome as a peptide-cleaving enzyme, which can be analysed by methods traditionally applied in single-enzyme kinetics. However, such an approach ignored the fact that the proteasome represents a whole kinetic system, which encompasses several spatial transport steps, as well as a combination of different enzymatic activities. Thus, quantifying the rate with which a model substrate is cleaved by the proteasome by measuring the appearance of cleavable products in the external space integrates across the contribution of different kinetic processes. With currently available experimental techniques, these processes cannot be individually dissected.

## 4. Modelling the Proteasomal Hydrolysis of Oligo- and Poly-Peptides

Whereas the models described above focus on the kinetic parameter of the active sites and potential regulatory sites, a second class of models focuses on the length distribution of generated fragments from oligo- or poly-peptides. Since the latter scenario is more complex and the basic kinetic characteristics of the proteasome are still not well defined, these models tended to contain a phenomenological description of the peptide hydrolysis. Furthermore, for reasons of simplicity, more or less strong assumptions needed to be made in order to describe such a system. As we will show, some of these assumptions were later on disproven by experimental studies, thereby demanding the development of new updated mathematical models.

The first attempt to mathematically model the pattern of proteolytic fragments generated by 20S proteasomes from oligopeptides was in 2000 by Holzhütter and Kloetzel [38]. Kinetic equations were derived that described the time course of the average cleavage probabilities of peptide fragments based on a given substrate sequence. The first assumption was that only a single substrate could enter the proteasome chamber. A second substrate could only enter once the first substrate was fully processed and all resulting fragments were released. However, this assumption has to be considered with care. Indeed, plasmon-resonance studies of Hutschenreiter *et al.* [39] revealed that the proteasome can bind two substrate molecules in a positive-cooperative manner. From this observation, the authors concluded that substrate cleavage might take place simultaneously in both half-proteasomes. Moreover, cryo-microscopic studies of Sharon *et al.* [33] suggested that all cavities of the 20S proteasome can simultaneously harbour bulky substrates, like green fluorescent protein or Cytochrome C. Furthermore, by investigating proteasomal peptide splicing, two groups showed that the proteasome can ligate together two peptides derived from different molecules, thereby demonstrating that a second substrate molecule can access proteasome cavity when the degradation products of the first molecule are still inside the cavity [8,40]. With the proposed stop-and-go process, Holzhütter and Kloetzel [38] also avoided having to include binding competition effects into their model. Furthermore, it was assumed that the affinity of a substrate only depends on the P1 residue for the first cleavage rather than the P1’ and surrounding residues. Finally, due to a lack of experimental evidence of substrate translocation rates, an exponential decay for the size dependency for the rate by which fragments are released was chosen. Despite all these assumptions, after calibrating the model to experimental data, the authors found that fragments generated by two cleavages had an average length of 7–13 amino acids and that their C-terminus was cleaved first, followed by the cleavage at the N-terminus. Overall, it was shown that only 10% of all possible cleavage site combinations (combinations of amino acid positions in a given substrate) were actually used to generate double-cleavage fragments. This is due to differences in the cleavage rates of the P1 residues, as well as due to the accessibility of the N- and C-fragment of the substrate in order to form a peptide bond.

In 2002, Peters *et al.* [41] formulated a kinetic model that describes the time evolution of fragment formation based on *in vitro* digestions of polypeptides by 20S proteasomes purified from human T2 and T2.27 cell lines. The model summarized all details involved in peptide hydrolysis as a single step, which was described by the overall observed procession rate. The procession rate includes all events from peptide uptake up to the release of the generated products of that peptide and is comparable to the peptide turnover. The model could be seen as a Michaelis–Menten-type model with phenomenological parameters. In this study, it was assumed that the peptide degradation rate was monotonously increased with increasing peptide length. For long polypeptides and entire proteins, this assumption did not hold, which limited the model to oligopeptide studies. Furthermore, a cleavage probability had to be *a priori* assigned to each peptide bond in the substrate. It was assumed that all cleavages occurred independently of one another. The authors developed the “mass-balance method”, which is based on the principle that the number of molecules in a reaction system is preserved. The method was used to calibrate the kinetic model against mass-spectrometry data. This study was able to simulate the time course of peptide fragments that was in good correspondence with the experimentally determined time course of fragment abundance.

A set of cleavage probabilities for a given substrate and the length of the substrate characterized the latter two mathematical models [38,41]. In 2005, Luciani *et al.* [42] proposed a model, which built upon those two studies. Here, the model was fully characterized by the length of the substrate [42]. The main assumptions of the previous studies, however, remained comparable, which was the decrease of the peptide release rate with increasing peptide length and a preferential production of fragments with a length of approximately nine amino acids. The peptide degradation is described by a Michaelis–Menten-type model, where the maximum reaction rate (v_max_) is inversely proportional to the peptide length. As a result, the model describes the length distribution of generated fragments over time. Interestingly, this study investigated the effect of gate opening on the length distribution by using data obtained with open-channel mutant proteasomes [16]. It was shown that opening of the gate resulted in an increase of long fragments and a decrease of shorter fragments, due to the increased rate of influx and efflux.

In 2008, Mishto *et al.* [43] proposed a further development of the latter mathematical model. This contains more details on the substrate-peptide uptake and release by the proteasome, which again was dependent on the length of the substrate-peptide. In addition to the peptide length, the authors introduced the substrate site-specific cleavage strength to describe the frequency of the substrate cleavage-site usage based on experimental data measured by mass spectrometry (using the 20S proteasome from the lymphoblastoid cell line, LcL). The model was calibrated against *in vitro* digestions of several polypeptide substrates. Another focus of this study was the effect of the proteasome regulator 11S complex on the time course of the hydrolysis products. The model outcomes suggested that the 11S complex increases the peptide uptake by the proteasome and allows a wider range of peptide lengths to be transported.

Several biophysical models have been proposed that describe the length distribution of peptide fragments. In these models, the active influx of peptide molecules into the proteasome chamber [44], as well as the translocation characteristics of peptides [45] were taken into account. A stochastic model to quantify the fragment patterns of polypeptide hydrolysis was developed later on by Goldobin *et al.* [46]. This model aimed at describing the protein translocation and cleavage, taking into account the topology and, thus, the reciprocal distance of the cleavage centres. The main steps modelled were the substrate translocation, substrate cleavage and product fragments removal. Substrate translocation was described as a sequence of residue-by-residue jumps induced by thermal noise. Jumps were allowed to occur only in the forward direction. The probability of a jump is assumed to depend on the length of the substrate. The translocation rate, therefore, only depends on the substrate length. By contrast, the cleavage of peptide bonds is described by residue-specific cleavage probabilities. The proteolytic fragments are assumed to be much more mobile than the substrate and, therefore, abandoned the proteasome chamber immediately after production. Any re-entry or competition effects were neglected.

Taken together, all published models dealing with the processing of oligopeptides need as input *a priori* information on strong and weak cleavage sites and the length distribution of fragments to come up with correct simulations of a limited set of *in vitro* digests.

## 5. Conclusions

The modelling approaches considered in this review aimed at elucidating specific aspects of the regulatory mechanisms controlling the rate and specificity of proteasomal peptide hydrolysis (Table 1). Based on kinetic modelling, it was suggested that the proteasome is a hysteretic enzyme complex [37], which undergoes substrate inhibition [15,24,37]. The (protein or peptide) substrate can also act as an activator by cooperative binding events [15,24]. This model-based prediction was later confirmed by the experimental studies of Kisselev *et al.* [25,26]. The length of the substrate was shown to impact the substrate uptake and translocation [38,44,46]. In addition to the regulatory effects exerted by the substrate itself, the mechanisms of further regulators were described. Indeed, Ritonavir was shown to enhance the hydrolysis by binding to a regulatory site and competitively inhibiting the hydrolysis by binding to the active site [24]. Mathematical modelling also allowed elucidation of the regulatory function of 11S in that this complex enhances the overall proteolytic activity without affecting the maximum activities.

**Table 1:** Overview of published mathematical models of proteasome peptide hydrolysis.

Type	Year	Author	Summary
short-fluorogenic peptide models	1996	Stein *et al.* [37]	proteasome model of enzyme hysteresis and substrate inhibition
	2000	Schmidtke *et al.* [24]	two-site modifier model of proteasome hydrolysis in the presence of effectors (Ritonavir)
	2000	Stohwasser *et al.* [15]	kinetic model of the effect of the activator 11S on proteasome hydrolysis
oligo- and poly-peptide models	2000	Holzhutter *et al.* [38]	time course of cleavage probabilities based on substrate sequence
	2002	Peters *et al.* [41]	time course of cleavage fragments based on substrate sequence
	2005	Luciani *et al.* [42]	time course of the fragment length distribution based on substrate length
	2008	Mishto *et al.* [43]	time course of the fragment length distribution based on substrate length and substrate-specific cleavage strength under the influence of the 11S activator
biophysical models	2006	Zaikin *et al.* [44]	biophysical model of the active influx of peptide molecules into the proteasome chamber
	2006	Zaikin *et al.* [45]	biophysical model of peptide translocation characteristics
	2009	Goldobin *et al.* [46]	stochastic model of protein translocation and cleavage

There are, however, some issues that should be taken into account before developing new mathematical models of proteasome and proteasome regulatory processes. For instance, short fluorogenic peptides can help us to gain an understanding of the detailed biochemical reactions inside the proteasome chamber. However, the information on the mechanism of proteasomal cleavage of peptide bonds obtained from kinetic studies with such small fluorogenic peptides is limited by the fact that several elementary processes with different kinetic features may contribute to the observed changes in the fluorescent intensity of the used chromophore (Figure 2). First, it remains unclear how the transport of peptides, and even smaller peptide-based compounds, such as the short fluorogenic substrates, between the external space and the interior of the proteasome influences the overall kinetics of substrate degradation. It is possible that the entry and release of peptides through the openings in the α-rings proceeds as a pure diffusion processes without significant interactions between the peptide and those amino acid residues shaping the surface of the openings. It cannot be excluded, however, that passage of the peptide through the openings involves one or even several distinct binding steps (peptide hopping) and that the affinity constants of these binding steps influence the measured overall proteasome affinity towards the peptide. Second, the experimental and mathematical studies [24,25,26] suggest the presence of non-catalytic binding sites, which, upon occupation by the peptide substrate, may alter the conformation of the proteasome and, thus, the kinetic properties of the catalytic sites. Third, none of the small reporter peptides in use are specific for one of the three different catalytic activities of the proteasome. Rather, it appears very likely that different active sites bind and cleave the small fluorogenic peptide with different efficiency [4], thus producing a complicated kinetics representing the superposition of the kinetics of each participating active site. At last, short fluorogenic peptides might have a weak unspecific binding to the inner or outer surface of the proteasome, leading to a depletion of the substrate available for degradation. The alteration of measured fluorescence can also derive from the use of different plate readers [47].

To overcome some of these limitations, it is crucial in our opinion to consider oligo- and poly-peptide studies. However, even for the 20S core proteasome, a mathematical model that can reliably reproduce the main proteolytic fragments generated from an arbitrary oligo-peptide or even full-length protein substrate does not exist so far. The main problem hampering the development of such a model is the lack of experimental information on the spatio-temporal processes that the peptide substrate undergoes inside the proteasome chambers. The application of fluorescence resonance energy for the determination of the approach of the peptide substrate to distinct sites within the proteasome chambers could help to reconstruct the reaction path in space and time [48]. In addition, the development of new algorithms for the absolute quantification of peptides, produced by *in vitro* digestion of polypeptides, using mass spectrometry may allow one to review previous results and to improve the kinetic models [4,8].

From a methodological point of view, there is a need to better validate proposed models experimentally, as well as computationally. It would also be helpful to perform a model comparison to test alternative hypothesis. Furthermore, in the discussed studies, the models are based on data using different proteasome types (mouse, human, cell lines), which might lead to contradictory mechanisms and conclusions.

In summary, even though the influence of regulatory mechanisms (e.g., 19S, 11S, non-catalytic modifier sites) can be directly concluded from experimental data, only kinetic modelling can push our knowledge with a deeper insight into the cause of the observed effects. Although the mathematical models published so far helped the scientific community to research proteasome function, several break-through studies recently improved the knowledge of the catalytic and regulatory processes of proteasome isoforms [9,21,23,49,50,51]. The time might be ripe for the development of new types of mathematical models, aiming at describing proteasome activities and regulations *in toto*. 

## References

[1] Glickman M.H., Ciechanover A. (2002). The ubiquitin-proteasome proteolytic pathway: Destruction for the sake of construction. Physiol. Rev..

[2] Schwartz A.L., Ciechanover A. (2009). Targeting proteins for destruction by the ubiquitin system: Implications for human pathobiology. Annu. Rev. Pharmacol. Toxicol..

[3] Ebstein F., Kloetzel P.M., Kruger E., Seifert U. (2012). Emerging roles of immunoproteasomes beyond MHC class I antigen processing. Cell. Mol. Life Sci..

[4] Mishto M., Liepe J., Textoris-Taube K., Keller C., Henklein P., Weberruß M., Dahlmann B., Enenkel C., Voigt A., Kuckelkorn U. (2014). Proteasome isoforms exhibit only quantitative differences in cleavage and epitope generation.

[5] Ding Q., Cecarini V., Keller J.N. (2007). Interplay between protein synthesis and degradation in the CNS: Physiological and pathological implications. Trends Neurosci..

[6] Vigneron N., Stroobant V., Chapiro J., Ooms A., Degiovanni G., Morel S., van der Bruggen P., Boon T., van den Eynde B.J. (2004). An antigenic peptide produced by peptide splicing in the proteasome. Science.

[7] Liepe J., Mishto M., Textoris-Taube K., Janek K., Keller C., Henklein P., Kloetzel P.M., Zaikin A. (2010). The 20S proteasome splicing activity discovered by splicemet. PLoS Comput. Biol..

[8] Mishto M., Goede A., Taube K.T., Keller C., Janek K., Henklein P., Niewienda A., Kloss A., Gohlke S., Dahlmann B. (2012). Driving forces of proteasome-catalyzed peptide splicing in yeast and humans. Mol. Cell. Proteomics.

[9] Huber E.M., Basler M., Schwab R., Heinemeyer W., Kirk C.J., Groettrup M., Groll M. (2012). Immuno- and constitutive proteasome crystal structures reveal differences in substrate and inhibitor specificity. Cell.

[10] Groettrup M., Kirk C.J., Basler M. (2010). Proteasomes in immune cells: More than peptide producers?. Nat. Rev. Immunol..

[11] Mishto M., Bellavista E., Santoro A., Stolzing A., Ligorio C., Nacmias B., Spazzafumo L., Chiappelli M., Licastro F., Sorbi S. (2006). Immunoproteasome and LMP2 polymorphism in aged and alzheimer’s disease brains. Neurobiol. Aging.

[12] Bellavista E., Andreoli F., Parenti M.D., Martucci M., Santoro A., Salvioli S., Capri M., Baruzzi A., del Rio A., Franceschi C. (2013). Immunoproteasome in cancer and neuropathologies: A new therapeutic target?. Curr. Pharm. Des..

[13] Bellavista E., Santoro A., Galimberti D., Comi C., Luciani F., Mishto M. (2014). Current understanding on the role of standard and immunoproteasomes in inflammatory/immunological pathways of multiple sclerosis. Autoimmune Dis..

[14] Basler M., Mundt S., Muchamuel T., Moll C., Jiang J., Groettrup M., Kirk C.J. (2014). Inhibition of the immunoproteasome ameliorates experimental autoimmune encephalomyelitis. EMBO Mol. Med..

[15] Stohwasser R., Salzmann U., Giesebrecht J., Kloetzel P.M., Holzhutter H.G. (2000). Kinetic evidences for facilitation of peptide channelling by the proteasome activator PA28. Eur. J. Biochem..

[16] Kohler A., Cascio P., Leggett D.S., Woo K.M., Goldberg A.L., Finley D. (2001). The axial channel of the proteasome core particle is gated by the RPT2 atpase and controls both substrate entry and product release. Mol. Cell.

[17] Raule M., Cerruti F., Benaroudj N., Migotti R., Kikuchi J., Bachi A., Navon A., Dittmar G., Cascio P. (2014). PA28alphabeta reduces size and increases hydrophilicity of 20S immunoproteasome peptide products. Chem. Biol..

[18] Groettrup M., Ruppert T., Kuehn L., Seeger M., Standera S., Koszinowski U., Kloetzel P.M. (1995). The interferon-gamma-inducible 11 S regulator (PA28) and the LMP2/LMP7 subunits govern the peptide production by the 20S proteasome *in vitro*. J. Biol. Chem..

[19] Dick T.P., Ruppert T., Groettrup M., Kloetzel P.M., Kuehn L., Koszinowski U.H., Stevanovic S., Schild H., Rammensee H.G. (1996). Coordinated dual cleavages induced by the proteasome regulator PA28 lead to dominant MHC ligands. Cell.

[20] Emmerich N.P., Nussbaum A.K., Stevanovic S., Priemer M., Toes R.E., Rammensee H.G., Schild H. (2000). The human 26S and 20S proteasomes generate overlapping but different sets of peptide fragments from a model protein substrate. J. Biol. Chem..

[21] Ruschak A.M., Kay L.E. (2012). Proteasome allostery as a population shift between interchanging conformers. Proc. Natl. Acad. Sci. USA.

[22] Cascio P., Hilton C., Kisselev A.F., Rock K.L., Goldberg A.L. (2001). 26S proteasomes and immunoproteasomes produce mainly N-extended versions of an antigenic peptide. EMBO J..

[23] Osmulski P.A., Hochstrasser M., Gaczynska M. (2009). A tetrahedral transition state at the active sites of the 20S proteasome is coupled to opening of the alpha-ring channel. Structure.

[24] Schmidtke G., Emch S., Groettrup M., Holzhutter H.G. (2000). Evidence for the existence of a non-catalytic modifier site of peptide hydrolysis by the 20S proteasome. J. Biol. Chem..

[25] Kisselev A.F., Garcia-Calvo M., Overkleeft H.S., Peterson E., Pennington M.W., Ploegh H.L., Thornberry N.A., Goldberg A.L. (2003). The caspase-like sites of proteasomes, their substrate specificity, new inhibitors and substrates, and allosteric interactions with the trypsin-like sites. J. Biol. Chem..

[26] Kisselev A.F., Kaganovich D., Goldberg A.L. (2002). Binding of hydrophobic peptides to several non-catalytic sites promotes peptide hydrolysis by all active sites of 20S proteasomes. Evidence for peptide-induced channel opening in the alpha-rings. J. Biol. Chem..

[27] Bech-Otschir D., Helfrich A., Enenkel C., Consiglieri G., Seeger M., Holzhutter H.G., Dahlmann B., Kloetzel P.M. (2009). Polyubiquitin substrates allosterically activate their own degradation by the 26S proteasome. Nat. Struct. Mol. Biol..

[28] Erguler K., Stumpf M.P. (2011). Practical limits for reverse engineering of dynamical systems: A statistical analysis of sensitivity and parameter inferability in systems biology models. Mol. Biosyst..

[29] Toni T., Welch D., Strelkowa N., Ipsen A., Stumpf M.P. (2009). Approximate bayesian computation scheme for parameter inference and model selection in dynamical systems. J. R. Soc. Interface.

[30] Silk D., Kirk P.D., Barnes C.P., Toni T., Rose A., Moon S., Dallman M.J., Stumpf M.P. (2011). Designing attractive models via automated identification of chaotic and oscillatory dynamical regimes. Nat. Commun..

[31] Liepe J., Kirk P., Filippi S., Toni T., Barnes C.P., Stumpf M.P. (2014). A framework for parameter estimation and model selection from experimental data in systems biology using approximate bayesian computation. Nat. Protoc..

[32] Kirk P., Thorne T., Stumpf M.P. (2013). Model selection in systems and synthetic biology. Curr. Opin. Biotechnol..

[33] Sharon M., Witt S., Felderer K., Rockel B., Baumeister W., Robinson C.V. (2006). 20S proteasomes have the potential to keep substrates in store for continual degradation. J. Biol. Chem..

[34] Liepe J., Filippi S., Komorowski M., Stumpf M.P. (2013). Maximizing the information content of experiments in systems biology. PLoS Comput. Biol..

[35] Drovandi C.C., Pettitt A.N. (2013). Bayesian experimental design for models with intractable likelihoods. Biometrics.

[36] Busetto A.G., Hauser A., Krummenacher G., Sunnaker M., Dimopoulos S., Ong C.S., Stelling J., Buhmann J.M. (2013). Near-optimal experimental design for model selection in systems biology. Bioinformatics.

[37] Stein R.L., Melandri F., Dick L. (1996). Kinetic characterization of the chymotryptic activity of the 20S proteasome. Biochemistry.

[38] Holzhutter H.G., Kloetzel P.M. (2000). A kinetic model of vertebrate 20S proteasome accounting for the generation of major proteolytic fragments from oligomeric peptide substrates. Biophys. J..

[39] Hutschenreiter S., Tinazli A., Model K., Tampe R. (2004). Two-substrate association with the 20S proteasome at single-molecule level. EMBO J..

[40] Dalet A., Stroobant V., Vigneron N., van den Eynde B.J. (2010). Differences in the production of spliced antigenic peptides by the standard proteasome and the immunoproteasome. Eur. J. Immunol..

[41] Peters B., Janek K., Kuckelkorn U., Holzhutter H.G. (2002). Assessment of proteasomal cleavage probabilities from kinetic analysis of time-dependent product formation. J. Mol. Biol..

[42] Luciani F., Kesmir C., Mishto M., Or-Guil M., de Boer R.J. (2005). A mathematical model of protein degradation by the proteasome. Biophys. J..

[43] Mishto M., Luciani F., Holzhutter H.G., Bellavista E., Santoro A., Textoris-Taube K., Franceschi C., Kloetzel P.M., Zaikin A. (2008). Modeling the *in vitro* 20S proteasome activity: The effect of PA28-alphabeta and of the sequence and length of polypeptides on the degradation kinetics. J. Mol. Biol..

[44] Zaikin A., Mitra A., Goldobin D., Kurths J. (2006). Influence of transport rates on the protein degradation by the proteasome. Biophys. Rev. Lett..

[45] Zaikin A., Kurths J. (2006). Optimal length transportation hypothesis to model proteasome product size distribution. J. Biol. Phys..

[46] Goldobin D., Zaikin A. (2009). Towards quantitative prediction of proteasomal digestion patterns of proteins. J. Stat. Mech..

[47] Cui Z., Gilda J.E., Gomes A.V. (2014). Crude and purified proteasome activity assays are affected by type of microplate. Anal. Biochem..

[48] Park J.E., Wu Y., Carmony K.C., Miller Z., Sharma L.K., Lee D.M., Kim D.Y., Lee W., Kim K.B. (2014). A fret-based approach for identification of proteasome catalytic subunit composition. Mol. Biosyst..

[49] Matyskiela M.E., Lander G.C., Martin A. (2013). Conformational switching of the 26S proteasome enables substrate degradation. Nat. Struct. Mol. Biol..

[50] Lander G.C., Estrin E., Matyskiela M.E., Bashore C., Nogales E., Martin A. (2012). Complete subunit architecture of the proteasome regulatory particle. Nature.

[51] Da Fonseca P.C., He J., Morris E.P. (2012). Molecular model of the human 26S proteasome. Mol. Cell.

